# Development of a standard operating procedure and checklist for rapid sequence induction in the critically ill

**DOI:** 10.1186/s13049-014-0041-7

**Published:** 2014-09-11

**Authors:** Peter Brendon Sherren, Stephen Tricklebank, Guy Glover

**Affiliations:** Kings Health Partners, Department of Anaesthesia and Critical Care, Guy’s and St Thomas NHS Foundation Trust, London, SE1 9RT UK; Department of Anaesthesia, The Royal London hospital, Whitechapel road, London, E1 1BB UK

**Keywords:** Standard operating procedures, Care bundle, Checklist, Rapid sequence induction, Intubation

## Abstract

**Introduction:**

Rapid sequence induction (RSI) of critically ill patients outside of theatres is associated with a higher risk of hypoxia, cardiovascular collapse and death. In the prehospital and military environments, there is an increasing awareness of the benefits of standardised practice and checklists.

**Methods:**

We conducted a non-systematic review of literature pertaining to key components of RSI preparation and management. A standard operating procedure (SOP) for in-hospital RSI was developed based on this and experience from large teaching hospital anaesthesia and critical care departments.

**Results:**

The SOP consists of a RSI equipment set-up sheet, pre-RSI checklist and failed airway algorithm. The SOP should improve RSI preparation, crew resource management and first pass intubation success while minimising adverse events.

**Conclusion:**

Based on the presented literature, we believe the evidence is sufficient to recommend adoption of the core components in the suggested SOP. This standardised approach to RSI in the critically ill may reduce the current high incidence of adverse events and hopefully improve patient outcomes.

## Introduction

Critically ill patients requiring emergent airway management are at high risk of hypoxia and cardiovascular collapse due to a significant pathology, deranged physiology and iatrogenic causes [1-5]. When compared to the theatre setting, airway adverse events that result in death or brain damage are 30 and 60-fold more frequent in the Emergency Department (ED) and Intensive care unit (ICU), respectively [1]. The Fourth National Audit Project (NAP4) highlighted many potential issues with emergent airway management in the United Kingdom (UK), including inexperienced operators, inadequate equipment availability, poor planning and non-technical skills [6].

As a way of combating such issues, many clinicians have suggested the greater adoption of guidelines, checklists and standardised practice [1,6]. Intubation bundles have been shown to reduce immediate severe life-threatening complications associated with intubation of ICU patients [7]. In prehospital and military environments it is well recognised that the higher the acuity of the situation, the greater the need to remove individual procedural preferences and to adhere to a standard operating procedure (SOP) [8-11]. Use of standardised equipment preparations and checklists are vital to limit human error while improving team communication and patient safety [12,13]. In a clinically challenging and stressful environment, standardised equipment and patient preparation will liberate extra bandwidth to maintain situational awareness and facilitate focus on patient care.

As individuals, we may feel that our own practice is safe but we also have a responsibility to improve institutional practice and safety. In attempt to improve emergent airway management we conducted a non-systematic review and devised a SOP for rapid sequence induction (RSI) of the critically ill.

## Methods

### Background

Guy’s and St. Thomas’ NHS trust has a 70-bed critical care capacity split across a number of units with over twenty intensive care consultants. It also has a nationally commissioned severe respiratory failure centre with extracorporeal membrane oxygenation (ECMO) capabilities and dedicated retrieval service.

The authors undertook a prospective review of eighteen RSIs over a three week period on the intensive care units within the trust in November 2013. Of the patients undergoing RSI, 38.9% suffered an adverse event (unpublished data). Although this rate was comparable to those published in the literature [4], we felt it necessary to undertake this quality improvement initiative.

### SOP development

The primary goal of our SOP was to ensure the following during RSI of critically ill patients:

- ***Maximise first pass intubation success -*** Multiple attempts at intubation is associated with increased risk of a ‘Can’t Intubate Can’t Ventilate’ (CICV) scenario [2].
- ***No hypoxia*.**
- ***No hypotension or dysrhythmia*.**
- ***No awareness -*** Avoiding haemodynamic collapse and death is of greater importance than awareness in patients in extremis.

A non-systematic review of English literature relating to key components of RSI preparation and delivery in the critically ill was conducted. Components concentrated on included pre-RSI assessment; patient position; pre and peri-RSI oxygenation; haemodynamic optimisation; monitoring; equipment; induction and neuromuscular blocking drugs; briefing and post-intubation care.

The SOP comprised of an equipment setup sheet, checklist and critically ill airway algorithm. A manual describing how to use these components and relevant references was also developed.

The initial draft of the SOP was emailed to the critical care consultants and senior nurses, and was presented at the departmental clinical governance day. All components were adjusted following feedback accordingly prior to introduction.

### Implementing and use of the SOP

Education and training are essential to ensure the appropriate use and maximal benefit of a SOP introduction. Integration into the unit’s induction programme and regular low fidelity training in the actual working environment was deemed the most appropriate way to maximise the impact of the SOP.

**Figure 1:**
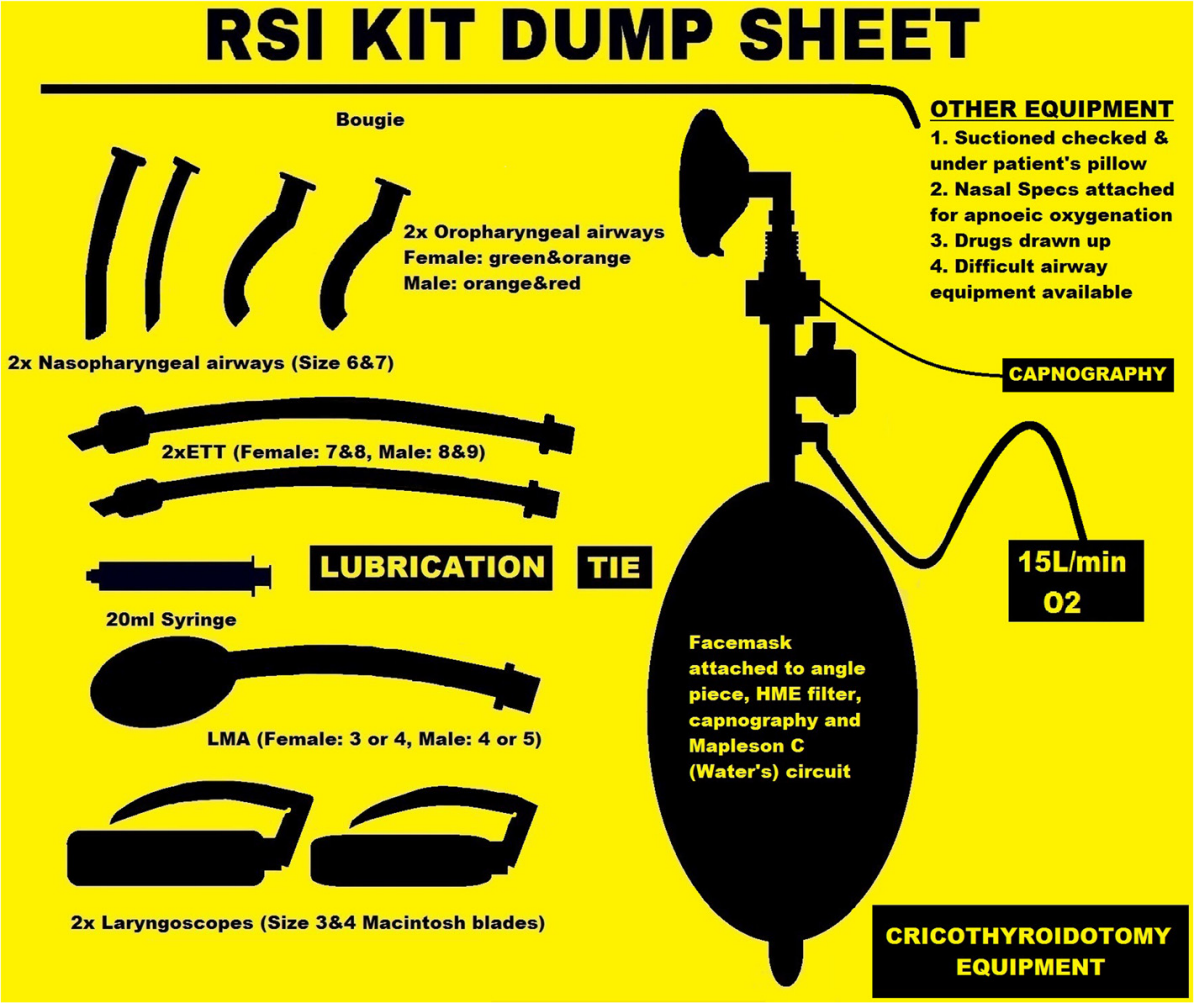
**Rapid sequence induction kit dump sheet.**

**Figure 2:**
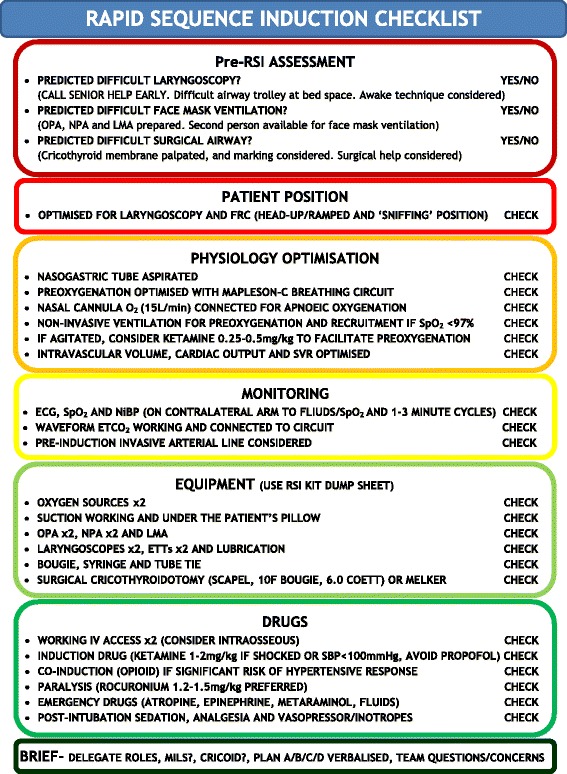
**Rapid sequence induction checklist.** OPA – oropharyngeal airway, NPA – nasopharyngeal airway, LMA – laryngeal mask airway, FRC – functional residual capacity, BVM – Bag-valve-mask, NRB – Non-rebreather, SVR – systemic vascular resistance, ECG – electrocardiogram, SpO_2_ – pulse oximetry, NiBP – non-invasive blood pressure, ETCO_2_ – End tidal capnography, ETT – endotracheal tube, MILS – manual in-line stabilisation.

Following appropriate introduction to the SOP and drilling during simulation, the SOP was designed to be used as follows. When a decision is taken to perform a RSI, members of the team should be allocated by the team leader to perform the following tasks simultaneously:

- Prepare equipment in a standardised fashion utilising the RSI kit dump sheet (Figure 1).
- Prepare the patient and monitoring according to the checklist (Figure 2).
- Prepare the RSI drugs, emergency drugs, fluids and post-intubation drugs.

Once completed, the airway doctor and a nominated team member should complete a final run through of the RSI checklist in a ‘challenge-and-response’ manner. For example the team member would ask “Patient position optimised for laryngoscopy and FRC” and the airway doctor response would be “CHECK”. This final cross-check can typically be completed during the preoxygenation period. Finally the team leader should deliver a brief to the entire team. The verbalised brief should include the airway plan, role allocation and allow time for any final concerns from the team, following which, the RSI can commence.

With appropriate training, the use of checklists should not delay the induction of anaesthesia [13].

## Rationale for the components of the checklist

### Pre-RSI assessment

All patients should be assessed for the likelihood of successful intubation, mask ventilation, supraglottic and surgical airway placement. There are a multitude of prediction tools available with limited evidence to advise definitively on one or another [14-16]. One of the most comprehensive and elegant prediction tools is advocated by *Walls* [14]:

- ***LEMON*** assessment for difficult laryngoscopy.
  - ○ ***L***ook externally - Beard, micrognathia, bull neck, buck teeth, facial trauma or airway bleeding.
  - ○ ***E***valuate with 3-3-2 rule - Inter-incisor distance <3 finger breadths, mental-hyoid distance <3 finger breadths, hyoid-thyroid notch distance <2 finger breadths.
  - ○ ***M***allampati - ≥3
  - ○ ***O***bstruction/***O***besity - Presence of any airway obstruction.
  - ○ ***N***eck immobility - Pathological, previous surgery or manual inline stabilisation.
- ***MOANS*** assessment for difficult facemask ventilation.
  - ○ ***M***ask seal inadequate.
  - ○ ***O***besity/obstruction.
  - ○ ***A***ge >55.
  - ○ ***N***o teeth.
  - ○ ***S***tiff/non-compliant lungs or ***S***leep apnoea.
- ***RODS*** assessment for difficult supraglottic airway insertion.
  - ○ ***R***estricted mouth opening.
  - ○ ***O***bstruction - Presence of any airway obstruction.
  - ○ ***D***istorted/***D***isrupted airway - Previous surgery, tumour or abscess.
  - ○ ***S***tiff/non-compliant lungs.
- ***SHORT*** assessment for difficult cricothyroidotomy.
  - ○ ***S***urgery previously to airway/neck or *S*hort laryngeal prominence to sternal notch distance.
  - ○ ***H***aematoma or abscess.
  - ○ ***O***besity.
  - ○ ***R***adiotherapy to neck previously.
  - ○ ***T***umour.

Following the appropriate assessment, extra equipment, senior help, surgical assistance or an awake technique may be appropriate. Awake fibreoptic intubations can be very challenging in patients with deranged physiology. Utilisation of such techniques should be reserved for experienced operators in an anticipated difficult intubation.

If difficult direct laryngoscopy or face mask ventilation is anticipated, the difficult airway trolley should be moved to the bed space. The likelihood of a failed intubation should heighten the degree of preparedness for rescue ventilation and a cricothyroidotomy. Depending on the likelihood of failed intubation and ventilation, a graded preparation for a cricothyroidotomy may be appropriate. This could be as simple as identifying and marking the cricothyroid membrane through to preparing the neck, epinephrine local infiltration and opening the surgical airway equipment prior to induction.

**Figure 3:**
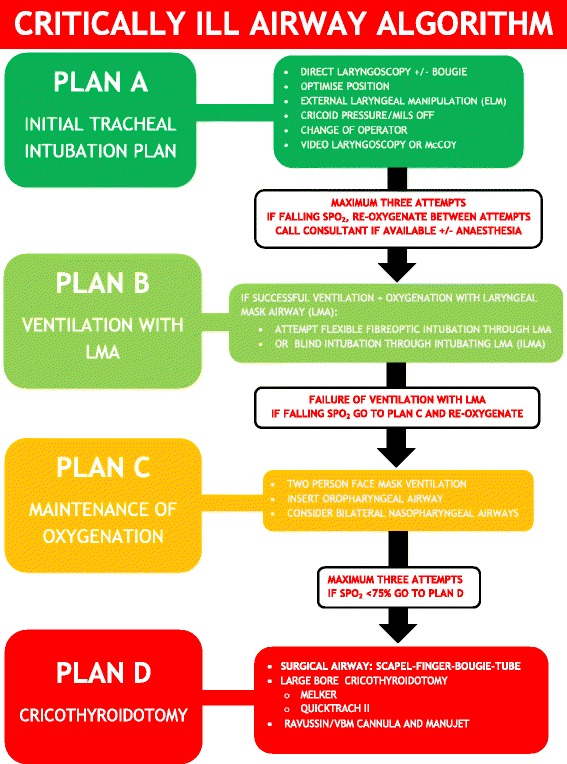
**Critically ill ‘traffic light’ airway algorithm.** MILS – manual in-line stabilisation, SpO_2_ – pulse oximetry.

Identifying the potential need for further help prior to induction is essential. The checklist and failed airway algorithm (Figures 2 and 3) should have the appropriate bleep numbers to contact if needed. If difficulty is anticipated, back-up personnel should be contacted before starting.

#### Summary

Assess ALL patients for the likelihood of difficult intubation, mask ventilation and surgical airway.

### Patient positioning

Patient positioning is crucial to maximise the functional residual capacity (FRC), total respiratory compliance and chance of successful intubation. Supine positioning reduces the FRC to a point where it may encroach on the closing capacity and result in increased atelectasis and shunting. This reduction in FRC and reservoir for preoxygenation has been shown to shorten the safe apnoeic time in elective anaesthesia [17,18]. This phenomena is only compounded by acute pulmonary pathology and is likely to limit the effectiveness of preoxygenation in the critically ill.

The head-up or ramped position has been shown to improve the FRC and safe apnoea time [17,18]. The head-up or ramped position may be particularly beneficial to oxygenation in obese patients [19]. Trauma patients with spinal precautions can still be preoxygenated in the reverse trendelenburg position to improve preoxygenation.

Optimisation of the head and neck in the ‘sniffing’ or ‘ear-to-sternal notch’ position is vital to ensure three axes alignment and optimal glottic visualisation [20]. Use of the ‘sniffing’ position along with 25° head-up has also been shown to improve laryngeal exposure, total respiratory compliance and the ease of ventilation [20-22].

#### Summary

Preoxygenate, induce, intubate and maintain anaesthesia in ALL critically ill patients in the ramped or 30° head-up position. Trauma patients should be managed in the reverse Trendelenburg position.

### Physiology optimisation

To optimise the safe apnoea period, all patients undergoing RSI should be preoxygenated for a minimum of three minutes via a high FiO_2_ source, during tidal-volume breathing [3,23]. In spontaneously breathing patients, a non-rebreather mask (NRBM) and self-inflating bag-valve-mask (BVM) may perform similarly in terms of denitrogenation, but a BVM is associated with an increased work of breathing [24]. However, the Mapleson-C breathing circuit is the most effective pre-oxygenation device and is better tolerated than BVM [25].

This type of preoxygenation is only likely to be effective if the patient has adequate spontaneous minute ventilation and no significant lung pathology or alveolar collapse. In the presence of a significant shunt, an ‘adequate’ SpO_2_ (≥97%) doesn’t necessarily represent sufficient denitrogenation of a diminished FRC. Although a Mapleson-C circuit can deliver CPAP, it may not represent the ideal preoxygenation device in patients with significant lung pathology and impaired respiratory drive. The use of non-invasive positive pressure ventilation preoxygenation has been shown to reduce severe hypoxemic episodes during the intubation of critically ill and obese patients [7,26,27]. In these trials, the use of NIV was not associated with any negative cardiovascular effects or obvious gastric distension [26,27]. Clinicians should have a low threshold for utilising NIV for preoxygenation in critically ill patients. NIV should be started with an PEEP 5-10cmH_2_O, FiO_2_ of 1.0, and then the inspiratory presure should be titrated to deliver tidal volumes of around 6-8 ml/kg.

Agitation and delirium are often cited as the reason for inadequate preoxygenation. Often the causes of poor cerebration are hypoxemia or hypercarbia, and bypassing preoxygenation to expedite the RSI may result in disastrous consequences. In this setting there is a role for procedural sedation to facilitate preoxygenation prior to RSI [28]. Delayed Sequence Intubation (DSI) describes the use of ketamine procedural sedation to facilitate preoxygenation with NIV [28]. Appropriate anxiolysis and sedation may also be achieved with suitable benzodiazepines and opiates. However, ketamine is particularly useful in this setting given its maintenance of airway reflexes and respiratory drive with a wide therapeutic margin [29]. Weingart’s essential paper gives a detailed description of DSI [28].

Non-critically ill patients consume approximately 250 ml of oxygen from the alveoli per minute. According to the respiratory quotient carbon dioxide production is 200 ml/min, however, only 8-20 ml/min moves into the alveoli during apnoea as the rest is buffered in the bloodstream [3,30]. This discrepancy results in a sub-atmospheric pressure within the alveoli during apnoea, and consequently entrainment of gas from the upper airway. This is known as apnoeic oxygenation, and is frequently employed during brainstem testing to avoid desaturation [31]. Apnoeic oxygenation can be achieved post neuromuscular blockade and during laryngoscopy with as little as 5 L/min of oxygen through nasal cannulae [32,33]. The low oxygen consumption during apnoea means that nasal cannulae are capable of delivering a high FiO_2_ within the pharynx [32]. Used in this way, apnoeic oxygenation can significantly prolong the time to desaturation by almost two minutes compared to standard care [32,33].

Every effort should be made to optimise the patient’s haemodynamics prior to any RSI. This will help mitigate the effects of drugs, loss of sympathetic outflow and positive pressure ventilation. Suitable fluid preloading should be accompanied by the appropriate use of inotropic and vasopressor agents. In the majority of cases, these drugs are best delivered through a central line and monitored with an invasive arterial line. In extremis, the delivery of fluids and ‘bolus dose’ inopressors through peripheral or intraosseous access may prove lifesaving.

#### Summary

Preoxygenate ALL patients for a minimum of three minutes, and have a low threshold for utilising NIV. Consider appropriate sedation if the patient is agitated and not complying with preoxygenation. Attach nasal cannulae running at 15 L/min to ALL patients during induction and laryngoscopy. ALL patients should have fluid connected with a pre-induction bolus if needed. Individualise vasopressors and inotropes as appropriate.

### Monitoring

Association of Anaesthetists of Great Britain and Ireland (AAGBI) compliant monitoring (ECG, SpO_2_, NiBP and ETCO_2_) should be used in all RSIs [34]. NiBP should be cycled every one to three minutes and a pre-induction BP should be seen. The blood pressure cuff should be placed on the opposite side to the pulse oximeter and the intravenous line being used for drug/fluid administration. Capnography is frequently underutilised in out-of-theatre intubations [4], despite recommendations from NAP4, AAGBI and the Intensive care Society [6,34,35]. Waveform capnography should be checked and connected to the circuit prior to induction for all RSIs.

Invasive arterial BP monitoring should be possible pre-induction in the majority of cases if needed. However, this must be balanced against the potential detrimental effects of delaying the RSI.

#### Summary

Use full AAGBI monitoring (ECG, SpO_2_, NiBP and ETCO_2_) for ALL out-of-theatre RSIs.

### Equipment

Difficult laryngoscopy is encountered in 11.3% of ICU intubations [5]; hence it is vital to prepare for failure in all critically ill RSIs with a minimum level of equipment always available. A standardised equipment setup (Figure 1) should be utilised for all out-of-theatres RSIs. The sheet will help limit confusion amongst team members on what is expected of them when setting up for a RSI. More equipment can be introduced as needed for expected difficult intubations. This equipment setup has been adapted and optimised from the authors’ own experience in various prehospital and retrieval medicine services but has minimal evidence base.

In addition to a standardised equipment setup, suction should be checked and placed under the patient’s pillow. Nasal cannulae for apnoeic oxygenation and appropriate preoxygenation device should also be applied.

#### Summary

There is a minimum level of equipment required for ALL emergent intubations. Utilise the RSI kit dump sheet (Figure 1) for ALL out-of-theatre intubations.

### Drugs

Anaesthetic agent choice for out-of-theatre RSI in the UK seems to be based largely on theatre based familiarity and not sound pharmacological principles [4]. Propofol and thiopentone are popular choices for RSI outside of the theatres [4]. In the critically ill, these induction agents may be safe in senior hands at 10-20% the normal dose in a controlled environment with invasive lines [36]. However, given their narrow therapeutic index and potentially disastrous haemodynamic collapse, propofol and thiopentone do not represent ideal induction agents [36-38].

The classic ‘cardiac anaesthetic’ with midazolam and high dose fentanyl has shown good haemodynamic stability in medically optimised patients [36]. In haemodynamically unstable patients, a very high endogenous sympathetic drive is vital to maintaining cardiac output and systemic vascular resistance. The use of fentanyl in such shocked patients can result in significant sympatholysis and hypotension [36,37,39]. Co-induction with fentanyl (1-3 mcg/kg) or alfentanil (10-20 mcg/kg) should only be considered in critically ill patients that are at high risk of a significant hypertensive response to laryngoscopy with detrimental consequences, such as patients with intracranial pathology.

Etomidate and ketamine fulfil many of the characteristics for an ideal induction agent in the critically ill [36,40]. Although a single dose of etomidate can inhibit endogenous steroid synthesis through its effects on 11β/17α hydroxylase, what is less clear is its impact on mortality [40-42]. Given the clinical equipoise, it would seem prudent to avoid etomidate in severe sepsis/septic shock [42]. Ketamine is a dissociative anaesthetic agent with sympathomimetic properties that results in a very desirable haemodynamic safety profile [36]. In many ways it is the ideal anaesthetic agent in the shocked patient [36]. There are many historical concerns over ketamine use, including its effect on intracranial pressure (ICP), most of which have now been disproved [36,43]. These concerns and lack of familiarity with its use, probably explains the limited use of ketamine within the UK [4]. The use of ketamine (1-2 mg/kg) is advocated as part of this SOP for the induction of shocked patients, or patients with a systolic blood pressure (SBP) less than 100 mmHg. Given ketamine can potentially increase blood pressure and myocardial oxygen demand, alternative agents should be considered in patients that are hypertensive, dysrhythmic or have acute myocardial ischaemia. For further information, Morris et al. recently published a detailed update on the use of ketamine as an induction agent [36].

High dose rocuronium (1.2-2.0 mg/kg) has been shown to offer similar speed of onset and intubating conditions when compared to suxamethonium for RSI [44,45]. Low cardiac output states may prolong the time to neuromuscular blockade, and 1.2 mg/kg of rocuronium should be regarded as the absolute minimum dose in critically ill patients for RSI [44,46].

The practicalities of ‘waking up’ a patient in a CICV scenario following suxamethonium administration are challenging and difficult to extrapolate to the critically ill population [46]. With the increasing availability of Sugammadex, this perceived benefit may only be of historical interest [46]. In addition, the longer duration of action of rocuronium ensures that optimal laryngoscopy and mask ventilation conditions are maintained when difficulties are encountered [46].

The increased oxygen consumption associated with suxamethonium fasciculations can also result in a significant reduction in the time to desaturation during apnoea when compared to rocuronium [47,48]. These issues combined with a desirable safety profile, makes rocuronium the obvious choice for paralysis during RSI [46].

#### Summary

Ketamine is the preferred induction agent in patients who are shocked or have SBP < 100 mmHg. Rocuronium is the preferred paralytic agent given its equivalent intubating conditions, desirable safety profile and prolonged time to desaturation during apnoea.

### Brief

Appropriate assessment and planning for failure were key deficits in care highlighted by NAP4 [6]. Planning must go beyond a simple internalised plan of what is likely to happen, and should include a comprehensive verbalised plan A, B, C and D. Verbalising the plan ensures a shared mental model and improves team dynamics.

The exact choice of laryngoscope and intubation plan is down to the individual clinician. A low threshold for the use of a bougie is advised to reduce the risk of repeated intubation attempts, particularly in patients with manual in-line stabilisation (MILS) [49,50]. Use of video-laryngoscopes and laryngeal mask airways may prove invaluable in difficult airway scenarios [49,51]. Whatever equipment is chosen, a structured failed airway plan should be verbalised and followed (Figure 3).

The use of cricoid pressure during emergent intubations remains controversial and has a minimal evidence base [52,53]. Outside of theatres it is often incorrectly applied and is known to increase the risk of failed intubation [53]. If deemed appropriate, cricoid pressure should only be applied by those familiar in its use, and if difficulty is experienced during laryngoscopy, there should be a low threshold for its removal. The use of bimanual external laryngeal manipulation on the other hand, may improve the grade of laryngoscopy and percentage of glottic opening [54].

Whenever a RSI is undertaken a well discussed plan should be formulated for a CICV scenario. Presence of the surgical airway equipment on the RSI kit dump sheet, and familiarity with the equipment and technique involved is essential. The low success rate of cannula versus surgical cricothroidotomy should be considered in a CICV scenario on the ICU [5,55-58]. A simplified scalpel-finger-bougie technique for surgical cricothroidotomy has been shown to have high success rates even with inexperienced operators [55,56].

Time for role allocation and an opportunity for the team to contribute any concerns or other issues should also be allowed prior to undertaking the RSI.

#### Summary

During ALL RSIs, the team leader should deliver a brief with the whole team present. They should verbalise a plan A/B/C/D, delegate roles and allow a final chance for any questions/concerns from the team.

### Post-intubation care

All tracheal intubations should have their position confirmed with waveform capnography in addition to auscultation [2,34,35]. Protective lung ventilation including tidal volumes of 6-8 ml/kg, plateau pressures less than 30cmH_2_0, optimal PEEP and titrated FiO_2_ should be initiated as soon as possible in all critically ill patients [59,60]. In patients with a neurological insult, neuro-protective ventilation with tight PaCO_2_ control (4.5-5kPa) and a PaO_2_ greater than 10kPa may be appropriate [61]. Patients with significant lung pathology are likely to de-recruit during RSI, this may necessitate early recruitment manoeuvres to minimise alveolar collapse and shunting. However, in haemodynamically unstable and hypovolaemic patients precautions should be taken to minimise tidal volumes and intrathoracic pressures to limit reductions in venous return and further cardiovascular collapse [11].

Early aggressive use of appropriate fluids and inopressors to improve tissue hypoperfusion is key to improving outcomes [62,63]. Physiological endpoints for resuscitation will depend on the underlying pathology [11,61-63].

Following long acting neuromuscular blockade it is vital to initiate appropriate sedation and analgesia early. The exact combination of medications will be dictated by patient haemodynamics, organ dysfunction and the likely period of ventilation required.

### Limitations

The idea of any SOP or care bundle is to amalgamate a collection of interventions based on best available evidence. Although not all components are based on a high grade of evidence, it is hoped that the cumulative effect of the interventions will improve the processes of care and patient outcomes. The limitations of this SOP are that it is based on variable grades of evidence from a non-systematic literature review, along with author and expert opinions. Although it is unlikely every clinician will agree with all constituents, the value of the presented SOP derives from the core components and package of care it provides.

## Conclusion

The use of the evidence based components of this SOP, along with the corresponding checklist and RSI kit dump sheet, will improve RSI planning, team dynamics and equipment availability. This standardised approach to RSI in the critically ill may reduce the incidence of adverse events and improve patient outcomes. Based on the presented literature we believe the evidence is sufficient to recommend adoption of the core points in the suggested SOP.
